# Study on the Aging Behavior of Natural Rubber/Butadiene Rubber (NR/BR) Blends Using a Parallel Spring Model

**DOI:** 10.3390/polym10060658

**Published:** 2018-06-12

**Authors:** Byungwoo Moon, Jongmin Lee, Soo Park, Chang-Sung Seok

**Affiliations:** Department of Mechanical Engineering, Sungkyunkwan University, Suwon-si, Gyeonggi-do 16419, Korea; moonmoon17@hanmir.com (B.M.); leepig87@gmail.com (J.L.); yabsabe@gmail.com (S.P.)

**Keywords:** NR/BR blends, crosslink density (CLD), strain-energy density (SED), aging behavior, prediction equation, swelling test

## Abstract

Natural rubber/butadiene rubber (NR/BR) blends are widely used in industrial areas for absorbing vibrations and shocks because of their excellent elastic stability. However, when an industrial-equipment surface is exposed to sunlight and oxygen over a long period of time, the rubber hardens. As a result, the tensile properties of the rubber material and the behavior of the strain-energy density function are changed, greatly reducing the performance of the rubber product. However, only a few experimental studies on the aging characteristics of NR/BR blends are available, and it is difficult to find a study that analyzes the organic relationship of the changes in the mechanical (stress–strain curves, strain-energy density, etc.) and chemical (cross-linked structure, crosslink density, etc.) properties. In this study, a swelling test was performed on an aged rubber compound, and the result was substituted into the Flory–Rehner equation to obtain the quantitative crosslink density. The results revealed a linear relationship between the strain-energy density (SED) and the crosslink density (CLD) when the cross-linked structure increase was represented by a parallel spring model. Finally, the relationship between the strain-energy density and the crosslink density was summarized as a formula, and a method for predicting the aging behavior of NR/BR blends using the crosslink density was proposed.

## 1. Introduction

Natural rubber–based blends, which have excellent resilience, are commonly used in industrial applications, e.g., tires and resistant products [1]. These types of blends are generally compounded by mixing natural rubber (NR) and butadiene rubber (BR) to satisfy both tensile and fatigue characteristics. However, rubber hardens when exposed to sunlight and oxygen for a long period of time [2]. As aging occurs, the tensile properties and the strain-energy density (SED) function of the rubber products change [3], greatly reducing the safety of industrial equipment, in contrast to the initial design conditions. The mechanical properties directly affect the performance of the rubber products, so research at the development stage is very important [4]. Therefore, in the design stage, it is important to evaluate the physical changes due to the aging characteristics.

In general, the exposure of rubber to real conditions for a long time is the most accurate method for evaluating the aging property; however, this is difficult to conduct realistically in terms of time and cost. Therefore, in most product-development stages, accelerated [5,6] test methods are mainly used, which are tested with aged rubber material under severe conditions. The physical-property change parameters for the aged rubber are typically evaluated using the hardness, tensile strength, permanent set, and elongation [7,8,9]. However, as the mechanical behavior is only apparent through experimental analysis and cannot demonstrate the fundamental reaction of the aged rubber, researchers have attempted to evaluate the aging characteristics using the crosslink density [10,11,12]. Furthermore, only a few experiments and tendencies were studied; studies analyzing the organic relationship of changes in the mechanical (stress–strain curves, strain-energy density, etc.) and chemical (cross-linked structure, crosslink density, etc.) properties are lacking.

In this study, a swelling test was performed to analyze the relationship between the strain-energy density, obtained by a mechanical experiment, and the crosslink density, obtained by a chemical experiment. The swelling test [13,14] results were substituted into the Flory–Rehner equation to obtain the crosslink density, and an equation assuming the cross-linked structure as a spring model was presented. Finally, the relationship between the strain-energy density and the crosslink density was summarized as a formula, and a method for predicting the aging behavior of NR/BR blends using the crosslink density was proposed.

## 2. Evaluation of the Swelling Properties of Aged Rubber

### 2.1. Swelling-Test Equipment and Method

The main material of the test specimen used in the swelling test was a compound containing 50% natural rubber (NR) and 50% butadiene rubber (BR). Accelerated aging was carried out using an environmental chamber (within 1 °C temperature error) to obtain the crosslink density of the rubber due to aging conditions (Table 1). The swelling specimen was 20 × 20 × 2 mm^3^ [15] and was weighed using a chemical balance. The solvent used in this test was a toluene (*V*_1_: 106.3 cm^3^/mol, $\rho_{toluene}$: 0.867 g/mL) [16,17] with a solubility constant of 8.91 (cal/cm^3^)^1/2^, suitable for the swelling reaction with the rubber. The specimens were completely immersed in the solvent for 72 h [17] at room temperature, and the wet weight was obtained when the equilibrium swelling was reached. Then, the swollen specimens were dried at room temperature for one day and weighed to obtain the dry weight [18,19]. 

The crosslink density ν was calculated by the Flory–Rehner Equation (1), where $M_{c}$ is the molecular weight of the rubber between crosslinks. $\nu_{ro}$ is the volume fraction of the swollen rubber, *V*_1_ is the molar volume of the solvent, and $\chi_{1}$ is the interaction coefficient indicating the reactivity between the polymer and the solvent.

(1) $$\nu =\frac{1}{2M_{c}}=-\frac{ln\left(1-\upsilon_{ro}\right)+\upsilon_{ro}+\chi_{1}\upsilon_{ro}{}^{2}}{2V_{1}\left(\upsilon_{ro}{}^{1/3}-\upsilon_{2}/2\right)}$$

Since the interaction coefficient of the polymer-solvent is a measure of how much the solvent dissolves in the polymer, the lower the value of $\chi_{1}$ is, the better the dissolution characteristics of the solvent into the polymer. The interaction coefficient between the NR rubber and the toluene is 0.393 [20,21,22], and the interaction coefficient between the BR and the toluene is 0.340 [23,24]. However, for the materials used in this study, NR and BR are blended compounds, so it is impossible to express them as one interaction coefficient. Therefore, the interaction coefficient ($\chi_{1}$) was calculated to be 0.367, using the experimental results for the change of the interaction coefficient with the solvent, depending on the NR/BR mixture ratio studied by Joseph et al. [25] (Table 2).

On the other hand, in a vulcanized rubber containing a fillers, e.g., carbon black, the volume fraction of the swollen rubber ($\nu_{ro}$) was determined from the Kraus Equation (2) [26].

(2) $$\frac{\nu_{ro}}{\nu_{rf}}=1-m\frac{\varphi }{1-\varphi }$$

where,

$$m=3c\left(1-\nu_{ro}{}^{\frac{1}{3}}\right)+\nu_{ro}-1\;$$

Here, φ is the volume fraction of the filler, *m* is a filler-rubber interaction parameter, and *c* is a parameter depending on the type of filler. $\nu_{rf}$ is the volume fraction [27,28] of rubber in the swollen filled rubber and is given by the following Equation (3)

(3) $$\nu_{rf}=\frac{\left(d-\varphi w\right)\rho_{p}{}^{-1}}{\left(d-\varphi w\right)\rho_{p}{}^{-1}+W_{t}\rho_{s}{}^{-1}}$$

where *d* is the weight of the sample after swelling and drying, φ is the volume fraction of the filler, *w* is the initial weight of the specimens, $\rho_{p}$ is the density of the polymer, $\rho_{s}$ is the density of the solvent, and $W_{t}$ is the amount of solvent absorbed specimen.

### 2.2. Swelling-Test Results

The crosslink density for the aged rubber is listed in Table 3. As the aging progressed, the swelling rate of the rubber specimens decreased. As a result, the unaged specimen expanded about 3.7 times as much as the initial volume; however, the specimen aged at 100 °C for 17 days was about 2.4 times larger than the initial volume. This is because, as the aging proceeds [29], the molecular weight of the rubber increases and chain molecules are generated, thereby widening the molecular-weight distribution. Thus, the rubber molecules have reduced flexibility [30], which limits the intermolecular slippage and reduces the swelling [31]. 

In the case of unaged specimens, the initial crosslink density is about 4.5 × 10^−5^ mol/cm^3^, and it can be confirmed that the crosslink density increases up to 14.5 × 10^−5^ mol/cm^3^ as the aging progresses. Moreover, even at the same temperatures of 70 °C and 80 °C, the crosslink density increased as the aging time increased from 17 days to 35 days. The results showed that the crosslinking density increased as the aging temperature and aging time increased, so the change of the quantitative properties of the aging rubber was explained using the results.

## 3. Evaluation of SED Functions of Aged Rubber

### 3.1. Relationship between CLD and SED

The metal material expresses the stress–strain relationship of the elastic section as *σ = E*∙*ε*, according to Hooke’s law. Thus, the elastic energy (resilience) per unit area can be expressed as $Res=\frac{E}{2}\cdot \varepsilon^{2}$, and the inherent elastic properties of the material can be expressed with Young’s modulus (*E*). However, in the case of rubber materials, the stress–strain relationship is nonlinear and the stress behavior of the rubber material varies, depending on the strain range. Therefore, it is necessary to obtain the elastic energy (strain-energy density) per unit area by integrating the lower area of the strain section with the nonlinear Simpson’s [32] rule. The strain-energy function can be formulated as an elongation function based on the Valanis–Landel [33,34] hypothesis. In this study, the strain-energy density function is expressed as $SED=k\cdot \varepsilon^{n}$, where *k* and *n* are rubber properties. 

**Figure 1:**
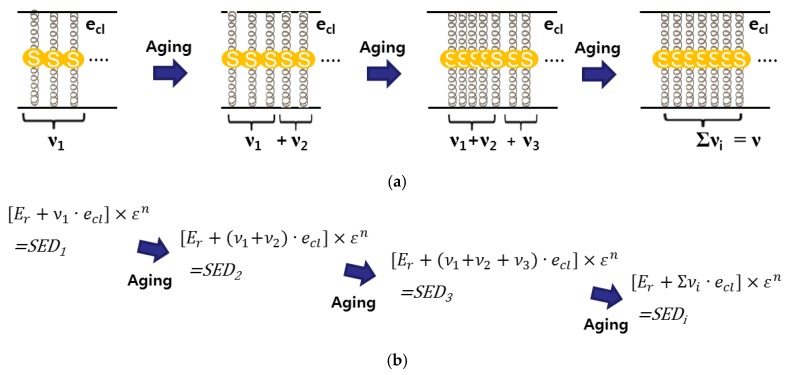
(**a**) Crosslink density expressed as a parallel spring model; (**b**) SED function expressed as a parallel spring model.

The coefficient of the strain-energy density function obtained from a previous study [35] is as shown in Table 4. Considering the change of the constant term (*k*) and the exponent term (*n*), the variation of the exponent term (*n*) was within 3%, but the constant term (*k*) increased up to 104% from the aging condition. To elucidate the relationship between the constant term (mechanical properties) and the crosslink density (chemical properties), which increases with aging, we assume that the increase in crosslink density due to aging follows the spring model, as shown in Figure 1a,b. 

**Figure 2:**
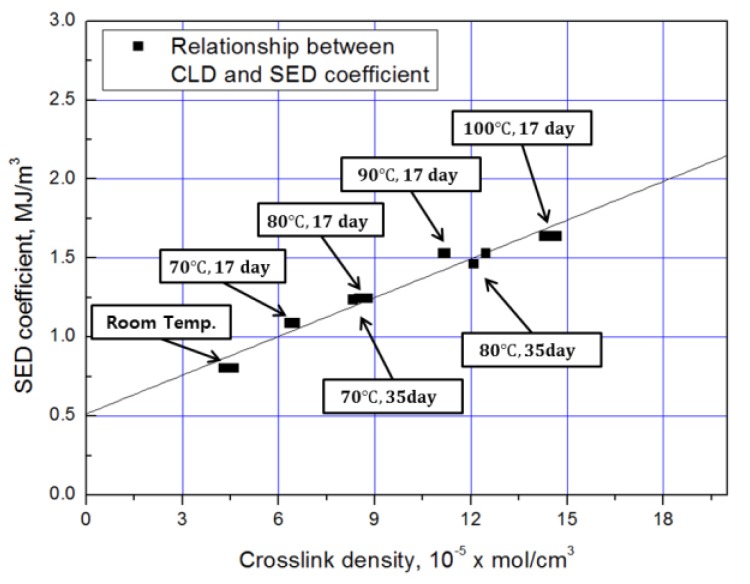
Relationship between CLD and SED function with constant (*k*).

The constant (*k*) of the strain-energy density function is expressed by (4), according to the spring model’s parallel-connection principle. We assume that $e_{cl}$ is the energy absorption coefficient per 1 mol of the cross-linked structure (MJ/mol), $E_{r}$ is the energy-absorption coefficient per unit area of the NR/BR blends (MJ/m^3^), and ν is the crosslink density. The relationship between the constant (*k*) of the obtained SED function and the crosslink density is shown in Figure 2. The energy-absorption coefficient per 1 mol of the cross-linked structure and the energy-absorption coefficient per unit area of the blends were obtained by fitting the equation for the spring model (4). When the constant (*k*) term is represented by a parallel spring model, the strain-energy density and the crosslink density are in a linear relationship, and the crosslink density increases as the aging progresses.

(4) $$k=E_{r}+e_{cl}\times \sum_{0}^{i}\upsilon_{i}$$

In this study, NR/BR blends energy-absorption coefficient $E_{r}$ and $e_{cl}$ are 0.514 MJ/m^3^ and 0.00818 MJ/mol as Equation (5).

(5) k=0.514+0.00818×υ

### 3.2. SED-Function Master Curve

In this study, we propose a master curve that can predict the behavior of the SED function, based on the results of previous experiments. By deriving a SED function with a specific aging temperature or aging time, we can predict its behavior under all conditions with the equivalent aging conversion using the Arrhenius Equation (6) [36,37,38,39], where *R* is a constant (8.314), *t* is a time, and *T* is the absolute temperature.

The exponent value (*n*) is selected as 1.69, using the average, and the constant value (*k*) affecting the SED function is fitted to the arc tangent and expressed as (7). Finally, the Arrhenius equation is substituted into (7) to derive the aged master curve as (8). In addition, by substituting (5), which shows the relationship between the crosslink density and the SED constant, into the strain-energy density function, the crosslink-density master curve is derived as (9).

(6) $$T_{1}=f_{1}\left(T_{2},\;t_{2}\right)=\frac{T_{2}\left(294.3\cdot ln\left(t_{2}\right)+292.2\right)}{1125-0.0867RT_{2}\left(2.83-ln\left(t_{2}\right)\right)}$$

$$f_{2}\left(T_{1}\right)=1.32-0.39\times {tan}^{-1}\left(\frac{355-T_{1}}{16}\right)$$

(7) $$SED=f_{2}\left(T_{1}\right)\cdot \varepsilon^{1.69}$$

(8) $$SED=\left[1.32-0.39\times tan^{-1}\left(\frac{355-f_{2}\left(T_{2},\;t_{2}\right)}{16}\right)\right]\cdot \varepsilon^{1.69}$$

(9) $$SED=\left[0.514+0.00818\times \upsilon \right]\cdot \varepsilon^{1.69}$$

## 4. Verification of Master Curves

We evaluated whether the change in the cross-linked structure occurs with the same mechanism at room temperature and an accelerated temperature, and thus obtained the crosslink density of rubber aged at room temperature (RT). The specimens were aged for two years in the shade with a temperature range of 8 ~ 25 °C in the laboratory. The average crosslink density of the unaged specimens was 4.5 × 10^−5^ mol/cm^3^, and the average crosslink density of the aged specimens at RT for two years was 6.0 × 10^−5^ mol/cm^3^. Table 5 confirms that the crosslink density of the aged specimen at room temperature increased by about 33.9%. 

**Figure 3:**
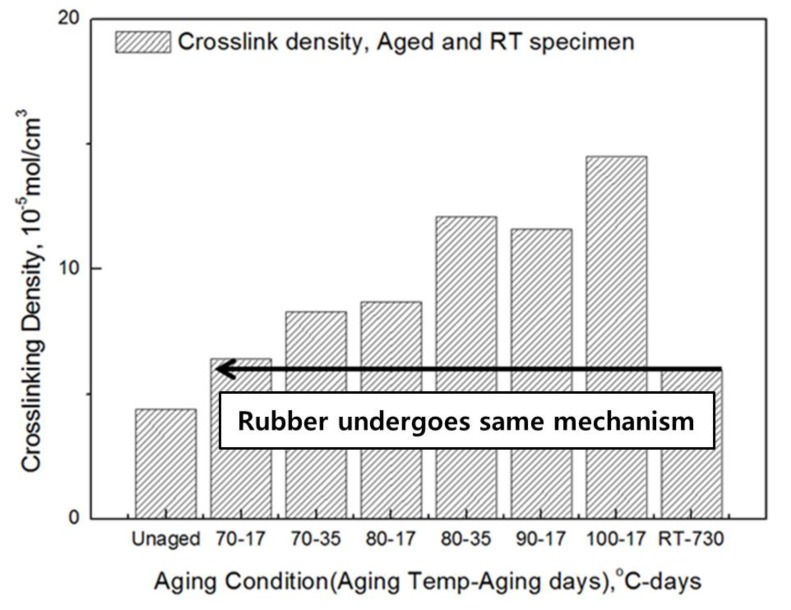
Comparison of crosslink density between aged and RT specimens.

The experimental values of the RT-aged specimens were compared with the aging specimens, and the RT-aged specimens were most similar to the specimens aged at 70 °C for 17 days (Figure 3). These results show that the aging speed differs between a long-term exposure at room temperature and a short-term exposure at a high temperature. However, the rubber molecule undergoes the same crosslinking-reaction mechanism, and it was confirmed that the crosslinking changes according to the aging condition. In the case of NR/BR blends, the thermal acceleration test derived the same results as the room temperature aging, and it was confirmed that the rubber properties due to aging affected the mechanical properties based on the chemical change (change of molecular bond).

**Figure 4:**
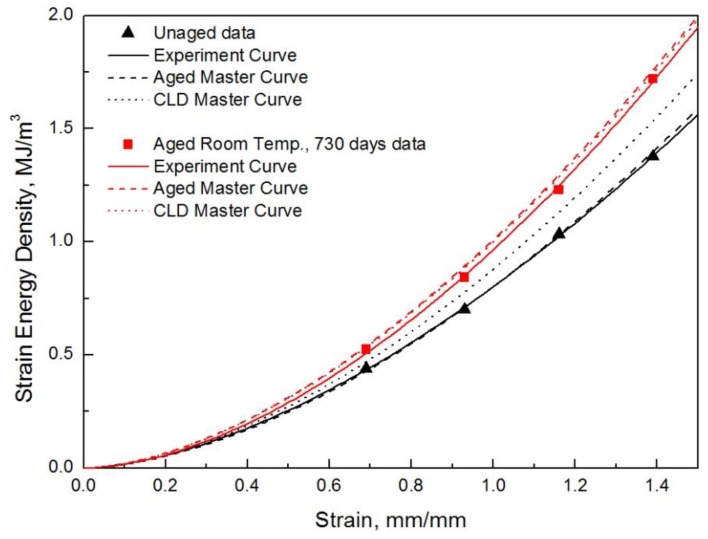
Master curve and validation.

Finally, we compared the experimental results with the predicted SED function using the master curve. The SED function was derived by substituting the average temperature (17 °C) and the number of days (365, 730 days) into the aged master curve (6). The crosslink density of the aged specimen at room temperature was substituted into the crosslink-density master curve (7) and the SED function was derived and compared. It was confirmed that the real test results were within 2.3% of the mean error when predicted using the aged master curve, as shown in Table 6, and within 5.8% of the mean error when predicted using the master density curve, as shown in Table 7 (Figure 4).

## 5. Conclusions

In this study, we analyzed the relationship between the crosslink-density increase and the behavior of the strain-energy density (SED) function with aging. In this process, the strain-energy density function was expressed by the crosslink density using a parallel spring model, and the following conclusions were obtained by comparing the test results with the predicted values.

1. A swelling test for accelerated-aged and room temperature-aged specimens was performed to obtain the crosslink density of the NR/BR blends. The results confirmed that the rubber properties affected the mechanical properties, based on the chemical change due to aging.
2. As the aging progressed, the strain-energy density and the crosslink density increased. Therefore, we assumed that the increase of the cross-linked structure was a parallel connection model to the spring, and a linear relationship was found between the strain-energy density and the crosslink density.
3. We proposed a method for predicting the aging characteristics of NR/BR blends using a swelling test, by summarizing the relation between the strain-energy density function and the crosslink density. By using this method, we derived an aged master curve that could obtain the behavior of the SED function according to the aging and the strain condition, and a crosslink-density master curve that could predict the behavior of the SED function by the swelling test.
4. When the experimental values and the predicted values were compared, it was confirmed that the strain-energy density value predicted using the aging master curve had a mean error of 2.3% or less, which was highly effective.
5. The tensile properties of rubber and the behavior of the strain-energy density function could be predicted by the aging. Therefore, it is possible to design in advance for the safety of mechanical equipment, tires, etc. In addition, the sustainability can be evaluated by predicting the SED by measuring the crosslink density of NR/BR blends.

## Figures and Tables

**Table 1:** Accelerated aging conditions of swelling specimens.

Aging Condition	Temperature, °C	Time, Days
Case 1	23	-
Case 2	70	17
Case 3	70	35
Case 4	80	17
Case 5	80	35
Case 6	90	17
Case 7	100	17

**Table 2:** Interaction coefficient of the polymer-solvent.

NR/BR Blend Ratio, phr *	100/0	80/20	60/40	50/50	40/60	20/80	0/100
**Interaction coefficient**, *χ*	0.393	0.382	0.372	0.367	0.361	0.351	0.340

* Parts per hundred rubber.

**Table 3:** Crosslink density (CLD) value according to the aging conditions.

Aging Conditions		#1	#2	#3	Average
Crosslink density (10^−5^ mol/cm^3^)	Unaged	4.3	4.6	4.4	4.4
	70 °C, 17 days	6.5	6.5	6.3	6.4
	70 °C, 35 days	8.3			8.3
	80 °C, 17 days	8.8	8.5	8.8	8.7
	80 °C, 35 days	12.1			12.1
	90 °C, 17 days	11.2	11.1	12.5	11.6
	100 °C, 17 days	14.7	14.4	14.3	14.5

**Table 4:** Change of SED function coefficient with aging.

Aging (°C, Days)	Normal	70, 17	80, 17	90, 17	100, 17
Coefficient number (*k*)	0.80	1.09	1.24	1.53	1.64
Exponential term (*n*)	1.65	1.69	1.71	1.71	1.70

**Table 5:** Crosslink density for room temperature–aged specimens.

Aging Condition		#1	#2	#3	Average
CLD (10^−5^ mol/cm^3^)	Unaged	4.3	4.6	4.4	4.5
	RT, 2 years	6.1	6.0	5.8	6.0

**Table 6:** Comparison of SED values according to experimental and aged master curves.

Aging Condition	Strain	Predicted SED	Test SED	Error, %
Unaged	0.69	0.43	0.44	2.7
	0.93	0.69	0.70	1.3
	1.16	1.07	1.03	2.7
	1.39	1.37	1.38	0.9
17 °C, 365 days	0.69	0.51	0.50	1.3
	0.93	0.84	0.83	1.9
	1.16	1.22	1.22	0.7
	1.39	1.66	1.67	0.4
17 °C, 730 days	0.69	0.53	0.53	2.6
	0.93	0.89	0.84	5.5
	1.16	1.30	1.23	5.2
	1.39	1.76	1.72	2.3
Standard error				2.3

**Table 7:** Comparison of SED values according to experimental and crosslink master curves.

Aging Condition	Strain	Predicted SED	Test SED	Error, %
Unaged	0.69	0.47	0.44	6.2
	0.93	0.77	0.70	9.1
	1.16	1.13	1.03	8.4
	1.39	1.53	1.38	9.8
17 °C, 730 days	0.69	0.54	0.53	1.9
	0.93	0.89	0.84	4.7
	1.16	1.29	1.23	4.5
	1.39	1.75	1.72	1.5
Standard error				5.8
